# Genome sequence of a traditional medicinal edible fungus *Calvatia gigantea* CGMCC5.9

**DOI:** 10.1128/mra.01036-23

**Published:** 2024-01-30

**Authors:** Yanhua Shi, Jinxin Chu, Weiping Lin, Junjie Li, Dianhai Hou, Lingjun Li

**Affiliations:** 1 Institution School of Bioscience and Technology, Weifang Medical University, Weifang, China; 2 School of Modern Agriculture and Environment, Weifang Institute of Technology, Weifang, China; University of California Riverside, USA

**Keywords:** fungal genome, *Calvatia gigantea*, *Lycoperdaceae*

## Abstract

The *Calvatia gigantea* is commonly used traditional medicinal edible fungus, which has multiple pharmacological effects. This paper reports the high-quality draft genome assembly of *Calvatia gigantea* CGMCC5.9, which consists of 39 scaffolds with 36.6 Mb (GC content, 48.37%), an N50 of 1,467,728 bp.

## ANNOUNCEMENT

The *Calvatia gigantea* belongs to the family *Lycoperdaceae* and is commonly used traditional medicinal edible fungus, which has multiple pharmacological effects, including antimicrobial, anti-inflammatory, anti-tussive, anti-cancer, and suppressing cell growth effects (1–5). Since little has been known about the genomic information of Lycoperdaceae species, here, we presented a high-quality draft genome assembly of *Calvatia gigantea* CGMCC5.9 obtained from the China General Microbiological Culture Collection Center (CGMCC) as a reference for future studies of this fungus. The mycelium of strain CGMCC5.9 was maintained on potato dextrose agar (PDA) medium (Solarbio, China) and then used as inoculum sequentially incubated in potato dextrose broth culture medium (Solarbio, China) (6).

Genome DNA was extracted from mycelia using DNeasy Plant Mini Kit (QIAGEN, Germany) as the kit protocol instruction and used for subsequent sequencing. A PacBio SMRTbell library was constructed and sequenced on a PacBio PacBio Sequel I platform at the ANORODA Biological Technology (Zhejiang) Co., Ltd. A paired-end Illumina library with an average insert size of 350 bp was constructed with the TruSeq DNA PCR-Free library preparation kit (Illumina, USA) and sequenced to produce 150 bp reads on an Illumina HiSeq X Ten system at the ANORODA Biological Technology (Zhejiang) Co., Ltd. Raw PacBio sequences were subjected to quality evaluation with the built-in built high-quality region finder and then were adapter trimmed and error corrected using Canu v1.8 (7), resulting in a high-quality long-read (>8 kb) data set of 1,091,681 PacBio reads (N50, 23,959 bp). Raw Illumina sequence reads were adapter and quality trimmed (8) using Cutadapt v2.6 (9) FastQC v0.11.9, resepctively, resulting in a high-quality short-read data set of 39,247,250 read pairs. Genome assembly was conducted by first using SmartDenovo v1.0 (10) with the PacBio clean-read data, refined using basic local alignment with successive refinement program v5.1 (11) with the parameter “--besttn5 --minMatch 18” and ARROW v2.2.1 for self-base correction, and further polished using Pilon v1.23 (12) with the Illumina clean-read data. Default parameters were used except where otherwise noted. The final assembled genome had a total length of 36.6 Mb (48.37% G + C content) composed of 39 scaffolds with a N50 of 1,467,728 bp and a mean read coverage of 102.48×. An internal transcribed spacer sequence from the genome sequence has been deposited in the NCBI GenBank (accession no. MW732168). Genome assembly quality was assessed with 82.8% of the complete BUSCOs (including 5.8% of Complete and duplicated BUSCOs) and 7.1% of fragmented BUSCOs using BUSCO v4.0 (13) aginst the Basidiomycota_odb9 data set (14).

## Data Availability

This whole-genome shotgun project has been deposited at GenBank under the accession number JACAAJ000000000. Raw Illumina and PacBio data have been deposited in the NCBI Sequence Read Archive under the BioProject accession number PRJNA640492.
